# Clipping versus coiling for treatment of middle cerebral artery aneurysms: a retrospective Italian multicenter experience

**DOI:** 10.1007/s10143-022-01822-3

**Published:** 2022-06-04

**Authors:** Carmelo Lucio Sturiale, Alba Scerrati, Luca Ricciardi, Oriela Rustemi, Anna Maria Auricchio, Nicolò Norri, Amedeo Piazza, Fabio Ranieri, Alberto Tomatis, Alessio Albanese, Vincenzo Di Egidio, Marco Farneti, Annunziato Mangiola, Enrico Marchese, Antonino Raco, Lorenzo Volpin, Gianluca Trevisi

**Affiliations:** 1 Department of Neurosurgery, Fondazione Policlinico Universitario A. Gemelli IRCCS, Università Cattolica del Sacro Cuore, L.go A. Gemelli 8 – 00168, Rome, Italy; 2 Department of Translational Medicine, University of Ferrara, Ferrara, Italy; 3 Department of Neurosurgery, Sant’Anna University Hospital of Ferrara, Ferrara, Italy; 4 NESMOS Department, Neurosurgical Unit, Sapienza University of Rome, Italy; 5 Department of Neurosurgery, San Bortolo Hospital, Vicenza, Italy; 6 Neurosurgical Unit, Ospedale Spirito Santo, Pescara, Italy; 7 Radiology Unit, Ospedale Spirito Santo, Pescara, Italy; 8 Department of Neurosciences, Imaging and Clinical Sciences, G. D’Annunzio University, Chieti-Pescara, Italy

**Keywords:** Middle cerebral artery, Intracranial aneurysm, Clipping, Coiling, Endovascular

## Abstract

Endovascular treatment has emerged as the predominant approach in intracranial aneurysms. However, surgical clipping is still considered the best treatment for middle cerebral artery (MCA) aneurysms in referral centers. Here we compared short- and long-term clinical and neuroradiological outcomes in patients with MCA aneurysms undergoing clipping or coiling in 5 Italian referral centers for cerebrovascular surgery. We retrospectively reviewed 411 consecutive patients admitted between 2015 and 2019 for ruptured and unruptured MCA aneurysm. Univariate and multivariate analyses of the association between demographic, clinical, and radiological parameters and ruptured status, type of surgical treatment, and clinical outcome at discharge and follow-up were performed. Clipping was performed in 340 (83%) cases, coiling in 71 (17%). Clipping was preferred in unruptured aneurysms and in those showing collateral branches originating from neck/dome. Surgery achieved a higher rate of complete occlusion at discharge and follow-up. Clipping and coiling showed no difference in clinical outcome in both ruptured and unruptured cases. In ruptured aneurysms age, presenting clinical status, intracerebral hematoma at onset, and treatment-related complications were significantly associated with outcome at both short- and long-term follow-up. The presence of collaterals/perforators originating from dome/neck of the aneurysms also worsened the short-term clinical outcome. In unruptured cases, only treatment-related complications such as ischemia and hydrocephalus were associated with poor outcome. Clipping still seems superior to coiling in providing better short- and long-term occlusion rates in MCA aneurysms, and at the same time, it appears as safe as coiling in terms of clinical outcome.

## Introduction

Thorough the last century, the gold standard for treating intracranial aneurysms (IAs) was microsurgical clipping until that the introduction of Guglielmi’s detachable coils (GDCs) in 1990 started a new chapter in the field of cerebrovascular surgery. Nevertheless, the first period of popularization of the endovascular technique led to significant controversies regarding the ideal management strategy for IAs, and the use of GDCs did not gain wide acceptance till the milestone publication of the International Subarachnoid Aneurysm Trial (ISAT) [1, 2]. Since then, the indications for coiling progressively refined and quickly became clear that not all IAs can be treated with GDCs as seen by a significant number of patients being excluded or crossed over from endovascular to surgical arms in some RCTs comparing the two techniques [1–3].

Aneurysm morphology and in particular wide neck or unfavorable dome-to-neck ratio as well as very small or very large size classically represent the less amenable angioarchitectural features for a definitive coiling entailing a higher risk of incomplete obliteration or recanalization.

Although these limitations have progressively reduced thanks to the increasing expertise and some technical refinements including balloon neck remodeling and stent assistance, clipping is still considered the best treatment for middle cerebral artery (MCA) aneurysms in many centers as it showed a higher safety and efficacy profile due to the specific anatomical characteristics of the MCA complex. In fact, despite MCA aneurysms are usually considered saccular in morphology, actually, they appear more similar to a bifurcation dysplasia, often with a large neck involving the origin of the collaterals, and their exclusion should be considered a bifurcation reconstruction rather than a simple aneurysm clipping. Their clipping have to guarantee at the same time exclusion of the sac, preservation of parent vessels ostia deformation, and perforators occlusion [4].

In this paper, we collected the recent experience of 5 Italian referral centers for cerebrovascular surgery on patients affected by MCA aneurysms undergoing surgical or endovascular treatment in order to compare short- and long-term clinical and neuroradiological outcomes.

## Materials and methods

### Population

We retrospectively reviewed 411 consecutive patients admitted to five Italian tertiary referral cerebrovascular centers between 2015 and 2019 with a diagnosis of ruptured or unruptured MCA aneurysms. All these patients underwent aneurysms securing through microsurgical clipping or endovascular coiling after the multidisciplinary cerebrovascular board consensus.

The selection of treatment for each case was based on characteristics of individual patients and aneurysms. In case of unruptured aneurysms, after the cerebrovascular board consensus, both modalities of treatment were presented to the patients and their preferences were also took into account. IRB approval was waived for this retrospective review of anonymous data.

### Inclusion/exclusion criteria

Inclusion criteria were age ≥ 18 year-old and availability of complete clinical and radiological reports. The cases of complex MCA aneurysms such as giant (> 25 mm) thrombosed and dissection were instead excluded from the study as considered special cases requiring complex and/or multiple interventions [5]. Finally, some few patients undergoing endovascular stenting or intra-aneurysmal devices releasing were excluded as they were not homogeneous by treatment with the vast majority of cases treated with coiling stand-alone. In case of patients with multiple aneurysms who underwent sequential treatments with multiple hospitalizations, we only consider that for the MCA aneurysm management.

By the analysis of all clinical and radiological reports, we retrieved the following:

1. *Patients characteristics*
  - Demographics: age, sex, and ethnicity
  - Hunt-Hess grading after SAH
  - Evidence of intracerebral hemorrhage (ICH) and/or hydrocephalus at presentation
  - Extended Glasgow outcome scale (eGOS) at discharge
  - eGOS and modified Rankin scale (mRS) grading at last follow-up
2. *Aneurysm characteristics:*
  - Size: maximum diameter; aspect ratio (aneurysm dome/neck diameter)
  - Morphology: surface irregularities, presence of a blebs; collaterals originating from aneurysm dome
  - Topography: side of MCA involvement
  - Ruptured status
  - Other: aneurysms multiplicity
3. *Treatment-related characteristics:*
  - Type of treatment (surgical clipping or endovascular coiling)
  - ICH removal
  - Hydrocephalus treatment (external ventricular drainage or ventriculo-peritoneal shunt)
  - Treatment-related complications
4. *Radiological characteristics:*
  - Early occlusion after treatment (complete, partial, failed)
  - Persistent occlusion/recanalization at follow-up (complete, partial, failed)
  - Clinically silent stroke images

### Surgical treatment

A standard pterional approach was performed in all cases of ruptured MCA aneurysms treated with clipping, while a minipterional craniotomy was adopted in 220 out of 248 (89%) unruptured aneurysms cases. Most of clipping procedures were performed with intraoperative monitoring of motor and somatosensory evoked potentials. In clipping cases, the patency of the parent arteries, major branches, and visible perforators was confirmed with micro-Doppler flowmetry and/or indocyanine green videoangiography.

### Endovascular treatment

All endovascular procedures were performed through direct aneurysm embolization with GDCs under general anesthesia, preceded by a diagnostic angiographic examination with 3D reconstructions. Biplane DSA was used instead of a single-plane according to the availability per center allowing simultaneous acquisitions of two projections thus reducing the number of contrast medium injections and procedural time and at the same time improving the spatial resolution of the exam.

### Outcomes analysis

Demographic, clinical, and radiological data were retrieved from patients’ medical records.

Radiological data were re-assessed for each patient with the help of expert neuroradiologists. All the available CT-angiography, MR-angiography, and DSA archived in the five institutional PACS systems were analyzed.

Clinical outcome was assessed at discharge and at follow-up and measured through the eGOS (upper good recovery, uGR; lower good recovery, lGR; upper moderate disability, uMD; lower moderate disability, lMD; upper severe disability, uSD; lower severe disability, lSD; vegetative state, VS; death, D) and the mRS. Telephone interviews were performed to retrieve missing and follow-up data.

### Outcomes comparison

Patients were dichotomized according to three main parameters: ruptured status, type of surgical treatment, and clinical outcome at discharge and follow-up. Then, we compared between the two groups the most important demographic, clinical, and radiological parameters in order to identify the significant differences. For patients with SAH, good outcome was considered eGOS upper/lower GR or upper/lower MD, whereas for patients treated for unruptured aneurysms, a good outcome was limited to eGOS upper or lower GR.

### Statistical analysis

Quantitative variables were expressed as mean ± standard deviation and the Student *t*-test was used to compare their means. If the equal variance assumption was violated, a Welch test instead of a Student’s test was used. Chi-squared test or, when more appropriate, the Fisher exact test (two-sided) were used to compare the categorical variables.

A multivariate analysis was performed through a binomial logistic regression model assuming clinical outcome at discharge and follow-up as depending variable and significant variables at univariate statistics as independent ones, distinguishing between ruptured and unruptured aneurysms.

Similarly, a multivariate model was developed to investigate those independent variables associated with radiological outcome dichotomized as complete or non-complete occlusion at both early control and follow-up.

Significance level was set at 0.05. Statistical analysis was performed using JASP 0.16.1.

## Results

### Comparison between patients treated with clipping and coiling

Demographic, clinical, and angioarchitectural characteristics of the included patients/aneurysms are reported in Table 1.

**Table 1:** Comparison between patients treated with clipping and coiling

		Unruptured aneurysms Total *n* = 248 (60%)			Ruptured aneurysms Total *n* = 163 (40%)			Ruptured + unruptured Total *n* = 411		
		Clipping *n* = 234 (94%)	Coiling *n* = 14 (6%)	*p*-value	Clipping *n* = 106 (65%)	Coiling *n* = 57 (35%)	*p*-value	Clipping *n* = 340 (83%)	Coiling *n* = 71 (17%)	*p*-value
Age ± SD in years		59 ± 10.5	55 ± 12.7	*ns*	55.7 ± 13.4	58.9 ± 13.6	*ns*	58.1 ± 11.5	58.2 ± 13.5	*ns*
Male sex		56 (24%)	7 (50%)	0.03	27 (25%)	15 (26%)	*ns*	83 (24%)	22 (31%)	*ns*
Topography	Right side	132 (56%)	8 (57%)	*ns*	61 (58%)	37 (65%)	*ns*	193 (57%)	45 (63%)	*ns*
	Left side	102 (44%)	6 (43%)		45 (42%)	20 (35%)		147 (43%)	26 (37%)	
Ruptured status	Ruptured	-	-	-	-	-	-	106 (31%)	57 (80%)	< .001
	Unruptured	-	-		-	-		234 (69%)	14 (20%)	
Mean size ± SD in mm		6.6 ± 4	9 ± 5.3	0.03	7.9 ± 4.2	7.2 ± 4.7	*ns*	7 ± 4.1 mm	7.6 ± 4.8 mm	*ns*
Mean AR ± SD		2.3 ± 3.4	2.2 ± 0.7	*ns*	2 ± 0.9	2 ± 0.6	*ns*	2.2 ± 2.4	2 ± 0.6	*ns*
Collaterals originating from aneurysm neck/dome		57 (24%)	2 (14%)	*ns*	24 (23%)	4 (7%)	0.01	81 (24%)	6 (8%)	0.004
Multiple aneurysms		103 (44%)	5 (36%)	*ns*	44 (42%)	14 (25%)	0.03	147 (43%)	19 (27%)	0.01
Blebs		65 (28%)	3 (21%)	*ns*	51 (58%)	20 (35%)	*ns*	116 (34%)	23 (32%)	*ns*
Hydrocephalus treatment	Transient EVD	-	-	-	27 (25%)	7 (12%)	0.04	27 (8%)	7 (10%)	*ns*
	Permanent shunt	5 (2%)	0	*ns*	13 (12%)	8 (14%)	*ns*	18 (5%)	8 (11%)	*ns*
Treatment-related neurological complications		19 (8%)	0	*ns*	21 (20%)	3 (5%)	0.01	40 (12%)	3 (4%)	*ns*
Clinically silent radiologic stroke		22 (9%)	1 (7%)	*ns*	7 (6.5%)	11 (19%)	0.01	29 (8.5%)	11 (15.5%)	*ns*
Treatment related general complications		10 (4%)	0	*ns*	14 (13%)	3 (5%)	*ns*	24 (7%)	3 (4%)	*ns*
Occlusion at discharge (total = 397)	complete	212 (92%)	10 (84%)	0.01	92 (91%)	37 (69%)	< .001	304 (92%)	47 (71%)	< .001
	partial	18 (8%)	2 (16%)	9 (9%)	17 (31%)	27 (8%)	19 (29%)			
	failed	0	0	0	0	0	0			
Occlusion at follow-up (total = 343)	complete	197 (92%)	9 (82%)	*ns*	72 (90%)	24 (63%)	< .001	268 (91%)	33 (67%)	< .001
	partial	18 (8%)	2 (18%)		8 (10%)	14 (37%)		26 (9%)	16 (33%)	
	failed	0	0		0	0		0	0	
Radiological follow-up in months (mean ± SD)		22.7 ± 16.6	28.2 ± 19.5	*ns*	26.4 ± 20	16.7 ± 9.4	< .001*	24 ± 17	19 ± 13	*ns*

Legend: *Welch’s unequal variances t test has been applied instead of Student’s t test as Levene’s test was significant (*p* < .05), suggesting a violation of the equal variance assumption; *AR*, aspect ratio

We included 411 patients treated for the same number of MCA aneurysms. Among them, 340 (83%) underwent clipping, whereas 71 (17%) underwent coiling stand-alone.

Considering the whole patients sample including patients with ruptured and unruptured aneurysms, no significant differences were observed in age, sex, aneurysms size, and topography between the two groups, whereas a significantly higher number of patients undergoing coiling were ruptured and most of those treated with surgery were unruptured. A dysplastic morphology, particularly when characterized by the presence of collateral branches originating from aneurysm neck/dome (21% out of cases), represented a key criteria to prefer a clipping reconstruction (*p* = 0.004).

An early radiological assessment after treatment was obtained in 397 cases. A complete occlusion was observed in 304 out of 331 patients (92%) undergoing clipping and in 47 out of 66 (71%) undergoing coiling (*p* < 0.001).

At the mean radiological follow-up of about 23 ± 17 months, which was obtained in 343 patients, a complete occlusion was still observed in 268 out of those undergoing clipping (91%), while it decreased at 33 out of 49 (67%) among those undergoing coiling (*p* < 0.001).

When separately considered patients with ruptured and unruptured aneurysms, some significant group-specific differences emerged especially among patients with ruptured aneurysms. In fact, a higher number of aneurysms with dysplastic morphology and unfavorable angioarchitectural features preferably underwent clipping. Likewise, the incidence of treatment-related neurological complications (symptomatic ischemia) appeared more common among patients with ruptured aneurysms undergoing clipping, whereas clinically silent radiologic strokes were more frequent among those underwent coiling. On the other hand, the occurrence of treatment-related general complications, such as wound infection and functional limitation during mastication as regards to patients undergoing operation, and dissection of femoral artery or femoral pseudoaneurysms for those underwent endovascular approach was not significantly different between the two groups.

Finally, as regards to the radiological outcome, the superiority in long-term occlusion rate observed among the aneurysms undergoing clipping was evident at follow-up only in the subgroup of ruptured aneurysm probably because these cases presented more complex morphological characteristics compared with the unruptured aneurysms group.

### Comparison between patients with ruptured and unruptured aneurysms

Patients were divided according to aneurysms ruptured status (Table 2). Among the 411 included patients, 248 (60%) harbored incidental unruptured aneurysms, while 163 (40%) had a ruptured MCA aneurysms with history of SAH at presentation.

**Table 2:** Comparison between ruptured and unruptured aneurysms

		Ruptured *n* = 163 (40%)	Unruptured *n* = 248 (60%)	Total *n* = 411	*p*-value
Age ± SD		57 ± 14	59 ± 11	58 ± 12	*ns*
Male sex		42 (27%)	63 (25%)	105 (26%)	*ns*
Topography	Right side	98 (60%)	140 (56%)	238 (58%)	*ns*
	Left side	65 (40%)	108 (44%)	173 (42%)	
Mean size ± SD		7.7 ± 4.4 mm	6.7 ± 4.1 mm	7.1 ± 4.3 mm	0.03
Mean AR ± SD		2.0 ± 0.9	2.2 ± 3.0	2.1 ± 2.2	*ns*
Collaterals originating from aneurysm neck/dome		28 (17%)	59 (24%)	87 (21%)	*ns*
Multiple aneurysms		58 (36%)	108 (44%)	166 (40%)	*ns*
Blebs		71 (43%)	68 (27%)	139 (34%)	< .001
Treatment	Clipping	106 (65%)	234 (94%)	340 (83%)	< .001
	Coiling	57 (35%)	14 (6%)	71 (17%)	
Treatment-related Complications		24 (15%)	19 (8%)	43 (10%)	0.02
Systemic complications		17 (10%)	10 (4%)	27 (7%)	0.01
Clinically silent radiologic stroke		18 (11%)	22 (9%)	40 (9.7%)	*ns*
Hydrocephalus treatment	Transient EVD	34 (21%)	0 (0%)	34 (8%)	< .001
	Permanent shunt	21 (13%)	5 (2%)	26 (6%)	< .001
eGOS at discharge	Upper GR	55 (33.7%)	233 (94%)	288 (70%)	< .001
	Lower GR	1 (0.6%)	1 (0.4%)	2 (0.5%)	
	Upper MD	32 (19.6%)	6 (2.4%)	38 (9.2%)	
	Lower MD	11 (6.7%)	0	11 (2.7%)	
	Upper SD	18 (11%)	6 (2.4%)	24 (5.8%)	
	Lower SD	17 (10.4%)	0	17 (4.1%)	
	VS	10 (6%)	1 (0.4%)	11 (2.7%)	
	D	19 (12%)	1 (0.4%)	20 (4.9%)	
eGOS at follow-up (total = 391)	Upper GR	60/144 (41.7%)	213/247 (86.2%)	273/391 (69.8%)	< .001
	Lower GR	19/144 (13.2%)	26/247 (10.5%)	45/391 (11.5%)	
	Upper MD	21/144 (14.6%)	4/247 (1.6%)	25/391 (6.4%)	
	Lower MD	0	0	0	
	Upper SD	26/144 (18.1%)	4/247 (1.6%)	30/391 (7.7%)	
	Lower SD	4/144 (2.8%)	0	4/391 (1%)	
	VS	9/144 (6%)	0	9/391 (2.3%)	
	D	5/144 (3%)	0	5/391 (1.3%)	
mRS at follow-up (total = 391)	0	60/144 (42%)	213/247 (86%)	273/391 (70%)	< .001
	1	19/144 (13%)	24/247 (10%)	43/391 (11%)	
	2	21/144 (15%)	5/247 (2%)	26/391 (7%)	
	3	16/144 (11%)	4/247 (1.6%)	20/391 (5%)	
	4	14/144 (10%)	1/247 (0.4%)	15/391 (4%)	
	5	9/144 (6%)	0	9/391 (2%)	
	6	5/144 (3%)	0	5/391 (1%)	
Mean ± SD clinical follow-up		23 ± 17 months	23 ± 16 months	23 ± 16 months	*ns*

Legend: *EVD* external ventricular drainage, *eGOS* Extended Glasgow Outcome Scale, *mRS* modified Rankin Scale, *AR* aspect ratio

The two groups were similar according to age, sex, and side distribution, whereas a significant difference was observable in terms of size as ruptured aneurysms were on average larger (7.7 ± 4.4 mm) and then unruptured ones (6.7 ± 4.1 mm). The frequency of blebs was also significantly higher in ruptured aneurysms (43% Vs 27%).

Patients with SAH had a median of Hunt-Hess score (IQR) at presentation of 3 ± 3 points. Among them, about 40% showed an associated intracerebral hemorrhage (ICH), which required evacuation in 32% out of cases.

A picture of hydrocephalus at presentation was present in about 65% of patients at the first CT-scan. Among them, about 34% needed an acute CSF diversion, which remained transient in 21% and was converted in a ventriculo-peritoneal shunt in 13%. The need of permanent shunts after clipping procedure occurred only in 2% among the unruptured MCA aneurysms (*p* < 0.001).

Similarly, the incidence of systemic (mainly infectious) and treatment-related (symptomatic strokes) complications appeared significantly higher in the ruptured aneurysms group, whereas the occurrence of the clinically silent radiologic strokes that was observed in about 10% out of cases was almost equally distributed between the two groups.

Surgery was in general the preferred treatment in 83% out of patients, with a significant prevalence among unruptured aneurysms compared with coiling (94% vs 65%), which was instead mainly adopted in case of ruptured aneurysms (35% vs 6%).

A global difference in clinical outcome at eGOS scale was recorded at discharge between the two groups, which was still observed at last mean follow-up of about 23 ± 16 months, at both eGOS and mRS scores.

### Comparison between patients with good and poor outcome

Patients were also dichotomized according to clinical outcome at discharge and follow-up differentiating between ruptured (Table 3) and unruptured (Table 4) aneurysms.

**Table 3:** Ruptured aneurysms: comparison between good and poor clinical outcome at discharge and at last follow-up according to Extended Glasgow Outcome Scale

		Good outcome at discharge *n* = 99 (61%)	Poor outcome at discharge *n* = 64 (39%)	Total *n* = 163	*p*-value	Good outcome at follow-up *n* = 100 (69%)	Poor outcome at follow-up *n* = 44 (31%)	Total *n* = 144	*p*-value
Age ± SD in years		54 ± 13.3	61.2 ± 12.8	56.8 ± 13.5	< .001	53.5 ± 12.4	60.3 ± 12.3	55.6 ± 13.4	0.01
Male sex		24 (24%)	18 (28%)	42 (26%)	*ns*	26 (26%)	12 (27%)	38 (26%)	*ns*
Topography	Right side	59 (60%)	39 (61%)	98 (60%)	*ns*	60 (60%)	26 (59%)	86 (60%)	*ns*
	Left side	40 (40%)	25 (39%)	65 (40%)		40 (40%)	18 (41%)	58 (40%)	
Mean size ± SD in mm		7.2 ± 4.4	8.4 ± 4.5	7.7 ± 4.4	*ns*	7.6 ± 4.5	7.7 ± 4.2	7.7 ± 4.4	*ns*
Mean AR ± SD		1.9 ± 1	2.2 ± 0.5	2 ± 0.8	*ns*	2 ± 1	2.1 ± 0.7	2 ± 0.9	*ns*
Collaterals from neck/dome		12 (12%)	16 (25%)	28 (17%)	0.03	14 (14%)	12 (27%)	26 (18%)	*ns*
Multiple aneurysms		36 (36%)	22 (34%)	58 (36%)	*ns*	36 (36%)	15 (34%)	51 (35%)	*ns*
Blebs		45 (45%)	26 (41%)	71 (44%)	*ns*	45 (45%)	22 (50%)	67 (47%)	*ns*
Mean Hunt-Hess score ± SD		2.4 ± 1.3	4 ± 1.2	3 ± 1.5	< .001	2.5 ± 1.3	3.75 ± 1.3	2.9 ± 1.4	< .001
ICH at presentation		35 (35%)	47 (73%)	82 (50%)	< .001	35 (35%)	33 (75%)	68 (47%)	< .001
Hydrocephalus at presentation		22 (22%)	19 (30%)	41 (25%)	*ns*	19 (19%)	15 (34%)	34 (24%)	*ns*
Treatment	Clipping	63 (64%)	43 (67%)	106 (65%)	*ns*	68 (68%)	29 (66%)	97 (67%)	*ns*
	Coiling	36 (36%)	21 (33%)	57 (35%)		32 (32%)	15 (34%)	47 (33%)	
ICH removal		18 (18%)	34 (53%)	52 (32%)	< .001	17 (17%)	24 (55%)	41 (28%)	< .001
Treatment-related Complications		3 (3%)	21 (33%)	24 (15%)	< .001	7 (7%)	10 (23%)	17 (12%)	0.007
Hydrocephalus treatment	EVD	18 (18%)	16 (25%)	34 (21%)	*ns*	16 (16%)	10 (23%)	26 (18%)	*ns*
	V-P shunt	13 (13%)	8 (12.5%)	21 (13%)	*ns*	11 (11%)	9 (20%)	20 (14%)	*ns*
Early Occlusion	Complete	76/93 (82%)	53/62 (85.5%)	129/155 (83%)	*ns*	80/96 (83%)	34/42 (81%)	114/138 (83%)	*ns*
	Partial	17/93 (18%)	9/62 (14.5%)	26/155 (17%)		16/96 (17%)	8/42 (19%)	24/138 (17%)	
Occlusion at follow-up	Complete	69/87 (79%)	27/31 (87%)	96 /118 (81%)	*ns*	71/90 (79%)	25/28 (89%)	96/118 (81.3%)	*ns*
	Partial	18/87 (21%)	4/31 (13%)	22 (19%)		19/90 (21%)	2/28 (11%)	22/118 (18.7%)	

Legend: Good outcome = upper or lower Good Recovery/ upper or lower moderate disability; poor outcome = upper or lower severe disability/vegetative state/death; *ICH*, intracerebral hemorrhage; *EVD*, external ventricular drainage; *V-P*, ventriculo-peritoneal; *AR*, aspect ratio

**Table 4:** Unruptured aneurysms: comparison between good and poor clinical outcome at discharge and at last follow-up according to Extended Glasgow Outcome Scale

		Good outcome at discharge *n* = 234 (94.4%)	Poor outcome at discharge *n* = 14 (5.6%)	Total *n* = 248	*p*-value	Good outcome at follow-up *n* = 239 (96.7%)	Poor outcome at follow-up *n* = 8 (3.2%)	Total *n* = 247	*p*-value
Age ± SD in years		58.7 ± 10.7	62.1 ± 9.3	58.9 ± 10.6	*ns*	58.7 ± 10.7	62.8 ± 7.6	58.9 ± 10.6	*ns*
Male sex		58 (25%)	5 (36%)	63 (25%)	*ns*	60 (25%)	3 (37.5%)	63 (25.5%)	*ns*
Topography	Right side	131 (56%)	9 (64%)	140 (56%)	*ns*	133 (56%)	6 (75%)	139 (56%)	*ns*
	Left side	103 (44%)	5 (36%)	108 (44%)		106 (44%)	2 (25%)	108 (44%)	
Mean size ± SD in mm		6.5 ± 3.1	10.2 ± 11.4	6.7 ± 4	ns*	6.7 ± 4.1	6.7 ± 3.9	6.7 ± 4.1	*ns*
Mean AR ± SD		2.3 ± 3.3	1.7 ± 0.6	2.2 ± 3.1	*ns*	2.3 ± 3.2	1.4 ± 0.6	2.3 ± 3.1	*ns*
Collaterals from neck/dome		54 (23%)	5 (36%)	59 (24%)	*ns*	54 (23%)	4 (50%)	58 (23%)	*ns*
Multiple aneurysms		102 (44%)	6 (43%)	108 (44%)	*ns*	103 (43%)	4 (50%)	107 (43%)	*ns*
Blebs		66 (28%)	2 (14%)	68 (27%)	*ns*	67 (28%)	1 (12.5%)	68 (28%)	*ns*
Treatment	Clipping	220 (94%)	14 (100%)	234 (94.4%)	*ns*	225 (94%)	8 (100%)	233 (94.3%)	*ns*
	Coiling	14 (16%)	0	14 (5.6%)		14 (6%)	0	14 (5.7%)	
Treatment-related complications		6 (2.6%)	13 (93%)	19 (7.7%)	< .001	10 (4.2%)	8 (100%)	18 (7.3%)	< .001
Hydrocephalus treatment	EVD	0	0	0	-	0	0	0	-
	V-P shunt	2 (0.8%)	3 (21%)	5 (2%)	< .001	2 (0.8%)	3 (37.5%)	5 (2%)	< .001
Early occlusion	Complete	209/228 (92%)	13/14 (93%)	222/242 (92%)	*ns*	214/233 (92%)	7/8 (87.5%)	221/241 (92%)	*ns*
	Partial	19/228 (8%)	1/14 (7%)	20/242 (8%)		19/233 (8%)	1/8 (12.5%)	20/241 (8%)	
Occlusion at follow-up	Complete	194/213 (91%)	12/13 (92%)	206/226 (91%)	*ns*	199/218 (91%)	7/8 (87.5%)	206/226 (91%)	*ns*
	Partial	19/213 (9%)	1/13 (8%)	20/226 (9%)		19/218 (9%)	1/8 (12.5%)	20/226 (9%)	

Legend: * Welch’s unequal variances *t* test has been applied instead of Student’s t test as Levene’s test was significant (*p* < .05), suggesting a violation of the equal variance assumption; good outcome = upper/lower good recovery; poor outcome = upper/lower moderate disability/upper/lower severe disability/vegetative state/death; *EVD*, external ventricular drainage; *V-P*, ventriculo-peritoneal; *AR*, aspect ratio

Among patients with ruptured aneurysms, those showing a good clinical outcome at discharge and follow-up were in general younger and showed a significantly worse clinical status (lower Hunt-Hess grading) after SAH.

Similarly, the occurrence of ICHs requiring evacuation and major treatment-related complications such as strokes was associated with a worse outcome both at short- and long-term follow-up.

On the other hand, no significant association among morphology, topography, and rate of occlusion was observed with clinical outcome, except for the presence of collaterals/perforators originating from aneurysms dome/neck, which increased the risk of ischemic complications and negatively influenced the short-term outcome (Table 3).

Among patients with unruptured aneurysms, the occurrence of treatment-related complications such as ischemia and hydrocephalus was the only reasons associated with a postoperative poor outcome both at discharge and at last follow-up that was on average of 23 ± 17 months (Table 4).

### Cosmetic and functional outcome

Overall personal satisfaction was high in our patients, and most of them emphasized that they were more psychologically relaxed since they had no more risk of aneurysm rupture. As regards the persistence of mastication disorders, most of patients treated with minipterional approach (196 out of 220; 89%) showed complete functional restoration with no masticatory pain at follow-up. On the other hand, 13 of 28 patients treated with standard pterional approach still complained of a slight pain during mastication and 7 had functional limitations in mouth opening at follow-up, compared with only 2 in the minipterional group.

From an esthetical point of view, a marked muscle temporal atrophy was rarely observed among patients approached with a minipterional craniotomy, and only 5% of them, which were pretty thin and with little hair, as well as about 20% of those treated with standard pterional craniotomy complained a subjective perception facial asymmetry.

### Multivariate analysis

According to our multivariate model (Table 5), older age, severity of clinical status at admission (Hunt-Hess), and occurrence of treatment-related complications were independently associated with poor outcome at discharge in patients with ruptured aneurysms, but the role of the complications (mainly ischemic) appeared no more significant at mean follow-up.

**Table 5:** Binomial logistic regression: outcomes (good or poor) according to extended Glasgow Outcome Scale

Dependent variable	Covariates	Odds ratio	*p*-value	95% confidence interval	
				Lower bound	Upper bound
Ruptured aneurysms					
*Outcome at discharge*	Age	1.061	0.002	1.022	1.102
	Hunt-Hess Score	2.029	< .001	1.454	2.832
	Collaterals (y)	2.816	0.093	0.843	9.411
	ICH (y)	1.849	0.295	0.585	5.841
	ICH surgery (y)	1.734	0.360	0.533	5.640
	Treatment related complications (y)	9.964	0.003	2.225	44.612
*Outcome at follow-up*	Age	1.066	< .001	1.030	1.104
	Hunt-Hess Score	1.748	< .001	1.279	2.389
	ICH (y)	1.946	0.235	0.649	5.835
	ICH surgery (y)	2.569	0.095	0.847	7.788
	Treatment related complications (y)	2.750	0.091	0.849	8.907
Unruptured aneurysms					
*Outcome at discharge*	Hydrocephalus treatment (shunt)	0.600	0.639	0.071	5.059
	Treatment related complications (y)	570.000	< .001	58.246	5578.088
*Outcome at follow-up*	Hydrocephalus treatment (shunt)	2.000	0.513	0.250	15.991
	Treatment related complications (y)	4.736e9	0.994	0.000	∞

Poor outcome coded as class 1. Poor outcome in ruptured aneurysms = upper and lower severe disability/vegetative state/death. Poor outcome in unruptured aneurysms = upper and lower moderate disability/severe disability/vegetative state/death

The occurrence of hydrocephalus as a treatment-related complication was instead the only variable independently associated with poor outcome at discharge, but not at follow-up, among patients treated for unruptured aneurysms.

Treatment was significantly associated with aneurysm obliteration at both early radiological control and at last follow-up (Table 6). Indeed, coiling had an odds ratio for non-complete obliteration of 247.5 (95% CI: 10.2–6008.4; *p* < 0.001) at early controls and of 155.157 (95% CI: 7.3–3309.2; *p* = 0.001) at last follow-up. Other factors associated with non-complete early obliteration were size and presence of collaterals, with size maintaining statistical significance at follow-up.

**Table 6:** Binomial logistic regression: radiological outcome (complete or non-complete obliteration) at early control and at last follow-up

Dependent variable	Covariates	Odds ratio	*p*-value	95% confidence interval	
				Lower bound	Upper bound
*Early obliteration*	Age	1.053	0.225	0.969	1.144
	Male gender	3.192	0.287	0.378	26.969
	Left side	0.476	0.475	0.062	3.644
	Hunt-Hess Score	1.552	0.240	0.745	3.235
	Size in mm	1.291	0.004*	1.087	1.534
	Aspect ratio	0.632	0.497	0.168	2.379
	Presence of collaterals	31.749	0.006*	2.739	367.992
	Presence of blebs	3.235	0.187	0.565	18.540
	ICH (y)	2.208	0.509	0.211	23.130
	Treatment (coiling)	247.548	< .001*	10.199	6008.422
*Obliteration at follow-up*	Age	1.078	0.092	0.988	1.176
	Male gender	1.908	0.465	0.337	10.792
	Left side	3.286	0.244	0.443	24.364
	Hunt-Hess Score	1.204	0.612	0.588	2.464
	Size in mm	1.229	0.004*	1.068	1.414
	Aspect ratio	0.758	0.603	0.266	2.159
	Presence of collaterals	3.181	0.286	0.379	26.703
	Presence of blebs	4.811	0.113	0.688	33.624
	ICH (y)	8.676	0.215	0.285	264.486
	Treatment (coiling)	155.157	0.001*	7.275	3309.179

Non-complete obliteration coded as class 1

Legend: * Statistically significant

## Discussion

In 2002, the ISAT results showed that the outcomes (absolute risk reduction in dependency or death) of endovascular coiling were significantly better than those of microsurgical treatment after 1 year of follow-up in cases of ruptured aneurysms [1].

This evidence progressively resulted in changing the preferred treatment for most of IAs from microsurgical clipping to endovascular coiling in several neurosurgical centers. Furthermore, despite the ISAT results were restricted to ruptured IAs and MCA aneurysms were underrepresented, the paradigm of “coiling first” gradually established regardless of aneurysms topography and the ruptured status.

However, though the advances in endovascular techniques observed over the last 20 years, surgical clipping still represents the gold standard for MCA aneurysm treatment in all experienced tertiary referral cerebrovascular centers, since several studies continue to support the superiority of open surgery in obtaining complete aneurysm occlusion with distal flow preservation, lower risk of perforator thromboembolism, and hemorrhagic infarction [6].

Moreover, lower risk of long-term recurrence and reduced probability of retreatment have also been associated with surgical clipping compared with coiling [7].

In agreement, in our multicenter series, about 83% of patients still underwent surgery, while coiling was preferred only in 17% out of cases, mostly ruptured.

Recently, a Middle Cerebral Artery Aneurysm Trial (MCAAT) started with the goal to compare results of clipping versus any endovascular management (including coiling, stent-assisted coiling and flow diversion) of ruptured and unruptured MCA aneurysms, while waiting for a randomized evidence, which is still missing [8]. MCAAT aims to provide a transparent care trial context for clinicians to optimally manage MCA aneurysms patients, by offering each one a 50% chance of being treated with clipping and a 50% chance of being treated with more recent, less-invasive but unproven endovascular options. In fact, the lack of convincing evidence in the neurovascular literature does not allow for rigid treatment protocols, and the choice of endovascular or surgical strategy is nowadays set accordingly to local expertise and clinical judgment.

From an anatomical point of view, despite MCA aneurysms are usually considered of saccular morphology, actually they are more similar to bifurcation dysplasia, often with large neck involving the origin of the main collaterals or small perforators. Accordingly, their treatment should be considered as a bifurcation reconstruction rather than a simple aneurysm clipping. During this complex reconstruction, exclusion of the sac, preservation from deformation of the parent vessels ostia, and avoidance of perforators occlusion have to be guaranteed at the same time adopting a fine dissection and respecting specific clipping rules [4, 9–11].

In our experience, the presence of collaterals originating from aneurysm neck/dome was in fact one the main reasons to favor open surgery in respect to coiling, especially in unruptured cases.

Nevertheless, as well as in peripheral hospitals, a gradual shift towards an endovascular approach for MCA aneurysms has also recently begun to be observed in larger referral centers. This is mainly due to two key-reasons: the progressive advances in endovascular instrumentations and the refinements in the technique, such as the balloon neck remodeling and stent assistance, in coiling procedures, as well as the parallel decline of the neurovascular expertise among neurosurgeons [12–15].

### Occlusion rate comparison between MCA aneurysms clipping and coiling

Johnston et al. in 2008 emphasized the importance of the complete sac obliteration for the risk of aneurysm re-rupture after SAH. Indeed, they found that cumulative re-rupture risk was 1.1% for complete occlusion, 2.9% for almost complete occlusion (91–99%), 5.9% for subtotal occlusion (70–90%), and 17.6% for partial occlusion (< 70%). Overall, for MCA aneurysms, the re-rupture risk tended to be greater after coiling than after clipping (3.4% vs. 1.3%, *p* = 0.092) because the rate of complete occlusion immediately after treatment was lower in the first groups and there was a higher rate of neck/sac partial refilling over time [16]. However, no cases of rebleeding were observed in our series along the entire period of follow-up.

A superior rate of complete occlusion with clipping was independently reported in 2011 by Güresir et al. [17] and van Dijk et al. [18] who described complete obliteration rates with clipping of 97.4% in 330 patients and of almost 90% in 107 patients with MCA aneurysms, compared with significantly lower rates (between 50 and 60%) obtained with coiling.

Similarly, in 2016, Choi et al. reported a complete obliteration in about 98% out of cases with clipping and about 56% with endovascular therapy in a series of 178 MCA aneurysms [19].

Finally, a comparative meta-analysis by Xin et al. published in 2019 found that surgical clipping of unruptured MCA aneurysms resulted in significantly higher complete aneurysms occlusion and lower incidence of retreatment and complications than coiling, concluding that surgery should be regarded as the first choice of treatment for unruptured MCA aneurysms [6].

In agreement with literature, in our series, we observed a rate of immediate complete obliteration of about 92% with surgery versus 71% with coiling. Furthermore, clipping group showed an occlusion rate stability at last follow-up, whereas about 5% of patients undergoing coiling presented partial aneurysm recurrence.

The reportedly complete obliteration rate of MCA aneurysms treated with coiling generally varies from 26.3 to 67% [12–14, 20, 21]. Therefore, our endovascular results settle on the higher rates of this trend. Anyway, the persistence of a higher prevalence of remnant necks in coiling compared to clipping appears difficult to overcome, due to the peculiar morphology of most of MCA aneurysms, characterized by a small dome-to-neck ratio, a relatively wide neck, and frequent incorporation of one of the distal branches in the aneurysm’s neck. Moreover, the new advanced techniques such as stent- and balloon-assisted embolization only partially compensate these disadvantages [22–24].

### Clinical and esthetic outcome comparison between MCA aneurysms clipping and coiling

A recent meta-analysis comparing the efficacy and safety of endovascular coiling versus microsurgical clipping for unruptured MCA aneurysms showed after a pooled analysis of 13 studies an unfavorable outcome in 2.1% (1.3–3.3%; 95% CI) of patients after clipping compared with 6.5% (4.5–9.3%; 95% CI) in 17 studies assessing the coiling technique [25].

This certain degree of discrepancy observed between the results of this meta-analysis and that of our series showing a poor neurological outcome at discharge in almost 6% of patients with unruptured MCA aneurysms and in 0% di coiling may have at least two possible explanations. First of all, in our series, the larger amount of included patients with unruptured aneurysms (more than 94%) were treated with clipping; secondly, we only considered good outcome patients with upper or lower GR at postoperative eGOS excluding MD patients.

A certain interest is also covered by the functional and aesthetic outcome. This appears reasonably pursuable in patients with unruptured aneurysms as those presenting with SAH are emergency cases with a life-threatening condition and the treatment has the main goal to be the safest rather than the most cosmetic.

For patients harboring unruptured aneurysms, instead, there is usually a preliminary agreement following a multidisciplinary discussion, which nowadays cannot avoid taking into account the patients demand for a more aesthetic approach.

While on the one hand, this request cannot justify the choice of endovascular treatment when this appears more risky in terms of safety, on the other, the refinement in the surgical technique and the miniaturization of the approach developed in the last few decades have responded effectively to this need for aesthetic results of patients.

### Limitations and perspectives

This study present several limitations: first, it has a retrospective design and has excluded some categories of MCA aneurysms such as the giant requiring more complex surgical treatments including bypass, and those treated of different endovascular devices. Moreover, a certain inhomogeneity in the indications to treat cannot be excluded among the different multidisciplinary boards as well as a different technical experience which may have influenced the final results.

Additionally, the results of our multicenter series only considered patients undergoing coiling stand-alone as the use of adjuncts was reported only in very few cases, which were therefore excluded from the present analysis. In fact, stents delivery in MCA aneurysms is a relatively recent extension of their indication as this technique presents several boundaries and procedure-related complications for this application. In fact, the presence of many small perforators may increase the possibility of thromboembolic events during the periprocedural period [23, 26, 27]. Also, a rare but potentially devastating complication is the delayed severe risk of in-stent stenosis, which needs patients should be closely followed-up over time [27, 28]. Accordingly, antiplatelets prophylaxis needs to be assumed for several months or lifelong when a stent-assisted coil embolization is performed. This reduces the possibility of their use in case of ruptured aneurysms, and in case of younger patients with unruptured MCA aneurysms who should intake antiplatelets for their entire life.

Nowadays, when conventional coiling is not possible despite a balloon or stent assistance, some new advanced endovascular devices such as intrasaccular flow-disruptors can represent an alternative option for MCA aneurysms [29–31], especially to meet the patients’ wishes of less invasive treatments.

However, there is still a low availability of clinical studies comparing clipping with advanced endovascular techniques for MCA aneurysms, but a recent study by Pflaeging et al. still showed that surgery is associated with a higher rate of complete occlusion and no additional morbidity, although in many cases endovascular treatment represented a safe and efficient alternative [32].

Lastly, our series includes a significantly higher number of clipped than coiled aneurysms. While this may induce some bias in the statistical analysis, it reflects the real-world attitude towards MCA aneurysms in 5 referral centers.

## Conclusions

MCA aneurysms are still largely considered from the neurosurgical community as having a higher chance of safe and effective treatment with clipping. However, the growing demand for less invasive treatments, the rapid spread of the endovascular techniques and their technical refinement, and the rapid increasing in operators experience impose a periodical reassessment of the comparison with the endovascular results. Our recent multicenter series confirms that clipping is not inferior to coiling in terms of clinical outcome, while continue to appear superior in terms of complete occlusion rate both at short- and long-term follow-up in ruptured and unruptured aneurysms.

## Data Availability

Available upon reasonable request.

## References

[1] Molyneux A, Kerr R, Stratton I, Sandercock P, Clarkes M, Shrimpton J, Holman R, International Subarachnoid Aneurysm Trial (ISAT) Collaborative Group (2002). International Subarachnoid Aneurysm Trial (ISAT) of neurosurgical clipping versus endovascular coiling in 2143 patients with ruptured intracranial aneurysms: a randomised trial. Lancet.

[2] Molyneux AJ, Kerr RS, Yu L-M, Clarke M, Sneade M, Yarnold JA, Sandercock P (2005). International subarachnoid aneurysm trial (ISAT) of neurosurgical clipping versus endovascular coiling in 2143 patients with ruptured intracranial aneurysms: a randomised comparison of effects on survival, dependency, seizures, rebleeding, subgroups, and aneurysm occlusion. The Lancet.

[3] Wiebers DO, Whisnant JP, Huston J, Meissner I, Brown RD, Piepgras DG, Forbes GS, Thielen K, Nicholas D, O’Fallon WM, Peacock J, Jaeger L, Kassell NF, Kongable-Beckman GL, Torner JC, International Study of Unruptured Intracranial Aneurysms Investigators (2003). Unruptured intracranial aneurysms: natural history, clinical outcome, and risks of surgical and endovascular treatment. Lancet.

[4] Sturiale CL, Rapisarda A, Marchese E, Puca A, Olivi A, Albanese A (2022). Surgical treatment of middle cerebral artery aneurysms: hints and precautions for young cerebrovascular surgeons. J Neurol Surg A Cent Eur Neurosurg.

[5] Scerrati A, Sabatino G, Della Pepa GM, Albanese A, Marchese E, Puca A, Olivi A, Sturiale CL (2019). Treatment and outcome of thrombosed aneurysms of the middle cerebral artery: institutional experience and a systematic review. Neurosurg Rev.

[6] Xin W-Q, Xin Q-Q, Yang X-Y (2019). Meta-analysis of clipping versus coiling for the treatment of unruptured middle cerebral artery aneurysms: direct comparison of procedure-related complications. Neuropsychiatr Dis Treat.

[7] Berro DH, L’Allinec V, Pasco-Papon A, Emery E, Berro M, Barbier C, Fournier H-D, Gaberel T (2020). Clip-first policy versus coil-first policy for the exclusion of middle cerebral artery aneurysms. J Neurosurg.

[8] Darsaut TE, Keough MB, Boisseau W, Findlay JM, Bojanowski MW, Chaalala C, Iancu D, Weill A, Roy D, Estrade L, Lejeune J-P, Januel A-C, Carlson AP, Sauvageau E, Al-Jehani H, Orlov K, Aldea S, Piotin M, Gaberel T, Gevry G, Raymond J (2022). Middle Cerebral Artery Aneurysm Trial (MCAAT): a randomized care trial comparing surgical and endovascular management of MCA aneurysm patients. World Neurosurg.

[9] Di Bonaventura R, Sturiale CL, Latour K, Mazzucchi E, Marchese E, Albanese A (2021). Comparison between minipterional craniotomy associated with focused Sylvian fissure opening and standard pterional approach with extended Sylvian fissure dissection for treatment of unruptured middle cerebral artery aneurysms. World Neurosurg.

[10] Sturiale CL, La Rocca G, Puca A, Fernandez E, Visocchi M, Marchese E, Sabatino G, Albanese A, Visocchi M, Mehdorn HM, Katayama Y, von Wild KRH (2017). Minipterional craniotomy for treatment of unruptured middle cerebral artery aneurysms. A single-center comparative analysis with standard pterional approach as regard to safety and efficacy of aneurysm clipping and the advantages of reconstruction. Trends in Reconstructive Neurosurgery.

[11] Tomatis A, Trevisi G, Boido B, Perez R, Benech CA (2019). Surgical outcomes and their correlation with increasing surgical experience in a series of 250 ruptured or unruptured aneurysms undergoing microsurgical clipping. World Neurosurg.

[12] Guglielmi G, Viñuela F, Duckwiler G, Jahan R, Cotroneo E, Gigli R (2008). Endovascular treatment of middle cerebral artery aneurysms: overall perioperative results. Apropos of 113 cases. Interv Neuroradiol.

[13] Kim BM, Kim DI, Park SI, Kim DJ, Suh SH, Won YS (2011). Coil embolization of unruptured middle cerebral artery aneurysms. Neurosurgery.

[14] Quadros RS, Gallas S, Noudel R, Rousseaux P, Pierot L (2007). Endovascular treatment of middle cerebral artery aneurysms as first option: a single center experience of 92 aneurysms. Am J Neuroradiol.

[15] Suzuki S, Tateshima S, Jahan R, Duckwiler GR, Murayama Y, Gonzalez NR, Viñuela F (2009). Endovascular treatment of middle cerebral artery aneurysms with detachable coils. Neurosurgery.

[16] Johnston SC, Dowd CF, Higashida RT, Lawton MT, Duckwiler GR, Gress DR, Investigators CARAT (2008). Predictors of rehemorrhage after treatment of ruptured intracranial aneurysms: the Cerebral Aneurysm Rerupture After Treatment (CARAT) study. Stroke.

[17] Güresir E, Schuss P, Berkefeld J, Vatter H, Seifert V (2011). Treatment results for complex middle cerebral artery aneurysms. a prospective single-center series. Acta Neurochir (Wien).

[18] van Dijk JMC, Groen RJM, Ter Laan M, Jeltema JR, Mooij JJA, Metzemaekers JDM (2011). Surgical clipping as the preferred treatment for aneurysms of the middle cerebral artery. Acta Neurochir (Wien).

[19] Choi JH, Park JE, Kim MJ, Kim BS, Shin YS (2016). Aneurysmal neck clipping as the primary treatment option for both ruptured and unruptured middle cerebral artery aneurysms. J Korean Neurosurg Soc.

[20] Biondi A, Janardhan V, Katz JM, Salvaggio K, Riina HA, Gobin YP (2007) Neuroform stent-assisted coil embolization of wide-neck intracranial aneurysms: strategies in stent deployment and midterm follow-up. Neurosurgery 61:460–468; discussion 468–469. 10.1227/01.NEU.0000290890.62201.A9 10.1227/01.NEU.0000290890.62201.A9 17881956

[21] Brinjikji W, Lanzino G, Cloft HJ, Rabinstein A, Kallmes DF (2011) Endovascular treatment of middle cerebral artery aneurysms: a systematic review and single-center series. Neurosurgery 68:397–402; discussion 402. 10.1227/NEU.0b013e318201d7f4 10.1227/NEU.0b013e318201d7f4 21135730

[22] Fields JD, Brambrink L, Dogan A, Helseth EK, Liu KC, Lee DS, Nesbit GM, Petersen BD, Barnwell SL (2013). Stent assisted coil embolization of unruptured middle cerebral artery aneurysms. J Neurointerv Surg.

[23] Regli L, Uske A, de Tribolet N (1999). Endovascular coil placement compared with surgical clipping for the treatment of unruptured middle cerebral artery aneurysms: a consecutive series. J Neurosurg.

[24] Yang P, Liu J, Huang Q, Zhao W, Hong B, Xu Y, Zhao R (2010). endovascular treatment of wide-neck middle cerebral artery aneurysms with stents: a review of 16 cases. AJNR Am J Neuroradiol.

[25] Smith TR, Cote DJ, Dasenbrock HH, Hamade YJ, Zammar SG, El Tecle NE, Batjer HH, Bendok BR (2015). Comparison of the efficacy and safety of endovascular coiling versus microsurgical clipping for unruptured middle cerebral artery aneurysms: a systematic review and meta-analysis. World Neurosurg.

[26] Yahia AM, Gordon V, Whapham J, Malek A, Steel J, Fessler RD (2008). Complications of Neuroform stent in endovascular treatment of intracranial aneurysms. Neurocrit Care.

[27] Yahia AM, Latorre J, Gordon V, Whapham J, Malek A, Fessler RD (2010). Thromboembolic events associated with Neuroform stent in endovascular treatment of intracranial aneurysms. J Neuroimaging.

[28] Fiorella D, Albuquerque FC, Deshmukh VR, McDougall CG (2004). In-stent stenosis as a delayed complication of neuroform stent-supported coil embolization of an incidental carotid terminus aneurysm. AJNR Am J Neuroradiol.

[29] Eboli P, Ryan RW, Alexander JE, Alexander MJ (2014). Evolving role of endovascular treatment for MCA bifurcation aneurysms: case series of 184 aneurysms and review of the literature. Neurol Res.

[30] Hagen F, Maurer CJ, Berlis A (2019). Endovascular treatment of unruptured MCA bifurcation aneurysms regardless of aneurysm morphology: short- and long-term follow-up. AJNR Am J Neuroradiol.

[31] Pierot L, Klisch J, Cognard C, Szikora I, Mine B, Kadziolka K, Sychra V, Gubucz I, Januel A-C, Lubicz B (2013). Endovascular WEB flow disruption in middle cerebral artery aneurysms. Neurosurgery.

[32] Pflaeging M, Kabbasch C, Schlamann M, Pennig L, Juenger ST, Grunz J-P, Timmer M, Brinker G, Goldbrunner R, Krischek B, Goertz L (2021). Microsurgical clipping versus advanced endovascular treatment of unruptured middle cerebral artery bifurcation aneurysms after a “coil-first” policy. World Neurosurg.

